# Two randomized controlled clinical trials to study the effectiveness of prednisolone treatment in preventing and restoring clinical nerve function loss in leprosy: the TENLEP study protocols

**DOI:** 10.1186/1471-2377-12-159

**Published:** 2012-12-18

**Authors:** Inge Wagenaar, Wim Brandsma, Erik Post, Wim van Brakel, Diana Lockwood, Peter Nicholls, Paul Saunderson, Cairns Smith, Einar Wilder-Smith, Jan Hendrik Richardus

**Affiliations:** 1 Erasmus MC, University Medical Center Rotterdam, Rotterdam, the Netherlands; 2 Independent Leprosy Consultant, Amsterdam, The Netherlands; 3 Royal Tropical Institute, Development Policy & Practice, Amsterdam, the Netherlands; 4 Department of Clinical Research, Faculty of Infectious and Tropical Diseases, London School of Hygiene and Tropical Medicine, London, UK; 5 Faculty of Health Sciences, University of Southampton, Southampton, United Kingdom; 6 American Leprosy Missions, Greenville, SC, USA; 7 Institute of Applied Health Sciences, School of Medicine and Dentistry, University of Aberdeen, Aberdeen, Scotland, UK; 8 Department of Neurology, Yong Loo Lin School of Medicine, National University of Singapore, Singapore, Singapore

**Keywords:** Leprosy, Prednisolone, Nerve function impairment, Subclinical neuropathy

## Abstract

**Background:**

Nerve damage in leprosy often causes disabilities and deformities. Prednisolone is used to treat nerve function impairment (NFI). However, optimal dose and duration of prednisolone treatment has not been established yet. Besides treating existing NFI it would be desirable to prevent NFI. Studies show that before NFI is clinically detectable, nerves often show subclinical damage. Within the ‘Treatment of Early Neuropathy in LEProsy’ (TENLEP) study two double blind randomized controlled trials (RCT) will be carried out: a trial to establish whether prednisolone treatment of 32 weeks duration is more effective than 20 weeks in restoring nerve function in leprosy patients with clinical NFI (Clinical trial) and a trial to determine whether prednisolone treatment of early sub-clinical NFI can prevent clinical NFI (Subclinical trial).

**Methods:**

Two RCTs with a follow up of 18 months will be conducted in six centers in Asia. In the Clinical trial leprosy patients with recent (< 6 months) clinical NFI, as determined by Monofilament Test and Voluntary Muscle Test, are included. The primary outcomes are the proportion of patients with restored or improved nerve function. In the Subclinical trial leprosy patients with subclinical neuropathy, as determined by Nerve Conduction Studies (NCS) and/or Warm Detection Threshold (WDT), and without any clinical signs of NFI are randomly allocated to a placebo group or treatment group receiving 20 weeks prednisolone. The primary outcome is the proportion of patients developing clinical NFI. Reliability and normative studies are carried out before the start of the trial.

**Discussion:**

This study is the first RCT testing a prednisolone regimen with a duration longer than 24 weeks. Also it is the first RCT assessing the effect of prednisolone in the prevention of clinical NFI in patients with established subclinical neuropathy. The TENLEP study will add to the current understanding of neuropathy due to leprosy and provide insight in the effectiveness of prednisolone on the prevention and recovery of NFI in leprosy patients. In this paper we present the research protocols for both Clinical and Subclinical trials and discuss the possible findings and implications.

**Trial registration:**

Netherlands Trial Register: NTR2300

Clinical Trial Registry India: CTRI/2011/09/002022

## Background

Damage to peripheral nerves is the main consequence of leprosy and may cause deformities and disabilities in patients. Nerve damage can occur before, during and after multidrug therapy (MDT) and is a result of inflammation in the nerves due to immunological reactions [1]. For many years corticosteroids, mostly prednisolone, have been used to treat nerve function impairment (NFI) in leprosy patients [2]. However, an optimal dose and duration of steroid treatment has yet to be established [3]. In addition, research should focus also on possibilities of timely detection and treatment of early (subclinical) neuropathy in order to prevent NFI and its consequences [4]. The TENLEP study is designed to obtain additional information about prednisolone treatment for preventing and restoring nerve function in people affected by leprosy. Within the TENLEP study two randomized clinical trials will be conducted; one trial focusing on patients with subclinical neuropathy, and the second trial focusing on patients with clinical NFI.

Between 6 and 27% of the 228 474 newly detected leprosy cases in 2010 [5] presented with visible impairment (grade 2 disability) [2,4]. This WHO leprosy disability grading system is the most widely used method to assess impairment in leprosy patients and is generally used for monitoring program quality [6,7]. More accurate assessments for NFI are Voluntary Muscle Testing (VMT) and Monofilament Testing (MFT) or ball point tests [8], which are widely used in clinical practice to assess motor and sensory NFI, respectively. Recently, more sensitive methods have been introduced to detect early, subclinical neuropathy. The INFIR study found that nerve conduction studies (NCS) and Warm Detection Threshold (WDT) are the most effective methods for finding subclinical nerve damage [1]. With these tests subclinical neuropathy can be detected at least 3 months before VMT and MFT can determine the first clinical impairments. Sub-clinical changes during and following MDT were also found to be predictive of new onset NFI [9].

To examine treatment of clinical NFI several studies have been conducted using prednisolone. In one cohort the WHO recommended prednisolone regimen (starting with 40 mg prednisolone/day, tapered down over 12 weeks [2]) was found not to be successful in the prevention and reversal of NFI in multibacillary (MB) patients treated for reactions or neuropathy [10]. Also a 16-week prednisolone regimen, usual practice nowadays, was found not to be very effective in two randomized controlled trials (RCT) [11,12]. One trial was in patients with Type 1 Reactions and/or NFI, receiving either prednisolone or prednisolone with intravenous methylprednisolone. Close to 50% of the patients required additional prednisolone during or after the treatment period [11]. The second RCT in MB patients with mild sensory impairment did not show a difference in NFI as measured with MFT 12 months after start of prednisolone treatment [12]. Van Veen et al. [3], reviewing RCTs comparing placebo to prednisolone treatment, conclude that studies so far have not provided enough evidence to draw robust conclusions about the long-term effect of corticosteroids on reactions and NFI. However, there is reason to assume that longer steroid treatment might be more beneficial, since one study showed that a 5 month corticosteroid regimen was significantly more effective than a 3 month regimen [13]. In TENLEP we aim to assess whether prednisolone treatment of 32 weeks duration is more effective than treatment of 20 weeks duration in restoring nerve function in patients with clinical sensory and/or motor NFI of recent onset (<6 mo), as detected by VMT and/or MFT.

The effect of prednisolone treatment on patients with subclinical neuropathy to prevent clinical NFI as determined with MFT and/or VMT has not been studied in a RCT before. The objective of the Subclinical trial is therefore to determine whether prednisolone treatment of early subclinical neuropathy, as detected with WDT and NCS, would prevent clinical sensory and/or motor function loss in leprosy patients. This paper presents the protocols for both the Clinical and the Subclinical trial.

## Methods

### Definitions

#### General

##### Neuropathy (peripheral)

Functional impairment and/or structural damage to autonomic, sensory, and motor nerve fibers within the peripheral nervous system.

##### Nerve function impairment

Sensory, motor or autonomic neuropathy evidenced by clinically detectable reduction in function in sensory, motor and/or autonomic fibers. The ‘level’ of impairment that is clinically detectable depends on the sensitivity of the testing instruments used. (It does not include abnormality of nerve conduction that is detectable only by electrophysiological means and WDT).

##### Nerve damage

An imprecise but common term for ‘neuropathy’, which is also used in relation to trauma. Here it indicates clinical or subclinical damage to a nerve, whether reversible or irreversible.

##### Neuritis

A condition in which inflammatory cells are found in the nerve, detectable by swelling and/or functional impairment with spontaneous nerve pain and/or nerve tenderness on palpation.

##### Subclinical neuropathy

Patients have normal values for Voluntary Muscle Testing and Monofilament Testing, but are impaired on Nerve Conduction Studies (NCS) and/or Warm Detection Threshold (WDT).

### Entry criteria Clinical trial

At least one nerve with either a VMT score of 4 or less on the 0–5 (modified) MRC scale or with an monofilament threshold increased compared to normal subjects by three or more monofilament levels on any test-site, two levels on one test-site AND at least one level on another test-site, OR one level on three or more test-sites for one nerve. With VMT each nerve is tested with one specific test assessing the strength of a muscle (group) innervated by that nerve. For MFT each nerve is tested on three sites).

### Entry criteria Subclinical trial

Any one parameter (Motor Nerve Conduction, Sensory Nerve Conduction, Warm Detection Threshold) abnormal in at least two nerves or any two parameters abnormal in at least one nerve. VMT and MFT values are normal.

### Outcome criteria Clinical trial

#### Restored nerve function

Monofilament Test and/or Voluntary Muscle Test of a nerve are recovered to normal levels (MFT = 0, VMT = 5).

#### Improved nerve function

At least one nerve shows better results on Monofilament Test and/or Voluntary Muscle Test. The MF thresholds should be *reduced* by three or more monofilament levels on any site, two levels on one site AND at least one level on another site, OR one level on three or more sites for one nerve. VMT score should be *increased* by at least 1 point.

#### Improved Reaction Severity Scale score

When the score on the Reaction Severity Scale [14]*decreases* by at least 3 points on the sum score or at least 2 points on any individual item in the scale.

#### Count of nerve function impairments (CNFI)

The sum of MFT and VMT scores (5 being normal) for all nerves tested in the study.

#### Improved SALSA score

The SALSA scale [15] score decreases at least with 1 category in standardized categories of SALSA values as described in SALSA Scale Users Manual (Salsa Scale Users Manual, Version 1.1, July 2010)

#### Improved participation scale score

The Participation scale [16] score decreases at least 1 grade on the “Grades of participation restriction” scale (as described on P-scale form).

### Outcome criteria subclinical trial

#### Clinical motor impairment (MI)

Motor neuropathy resulting in weakness of the muscles innervated by a given nerve. A patient is diagnosed as having clinical motor impairment if the VMT score for a muscle test is 4 or less on the 0–5 (modified) MRC scale. If a score of 4 is found, the test will be repeated by a second assessor.

#### Clinical sensory impairment (SI)

A patient is diagnosed as having sensory impairment of a nerve if the monofilament threshold is increased by three or more monofilament levels on any site, two levels on one site AND at least one level on another site, OR one level on three or more sites for one nerve.

#### Subclinical nerve function score (SNFS)

The SubClinical Nerve Function Score (SNFS) will be computed for NC parameters (amplitude and latency) and warm detection threshold, where non-impaired parameter adds 1 point to the overall score.

#### Improved SNFS

SubClinical Nerve Function Score increased with at least 1 point

#### Deteriorated SNFS

SubClinical Nerve Function Score decreased with at least 1 point

### Design of study

The TENLEP study consists of two multi-centre randomized triple blind controlled trials, both with two treatment arms, to study the effectiveness of prednisolone treatment restoring (Clinical trial) and in preventing (Subclinical trial) clinical nerve function loss.

Six institutions in four different countries participate in this study: Nepal (Lalgadh Hospital and Anandaban Hospital); India (JALMA Institute for Leprosy -Agra and Foundation for Medical Research-Mumbai); Bangladesh (Nilphamari Hospital); and Indonesia (Dr. Soetomo Hospital- Surabaya). Anandaban Hospital and Dr Soetomo Hospital will only take part in the Clinical trial. All collaborative institutions are referral hospitals specialized in the detection and treatment of leprosy. At each institution a Principal Investigator (PI) is responsible for the research at that centre. The overall responsibility lies with the International Coordinator (dr. E. Post), guided by an International Steering Committee.

#### Participants

In the Clinical trial leprosy patients with clinical NFI will be randomly allocated to either treatment of standard duration (20 weeks) or an interventional treatment of longer duration (32 weeks). Both multibacillary (MB) and paucibacillary (PB) patients diagnosed with clinical sensory and/or motor nerve impairment of less than six months duration are enrolled. For the Subclinical trial leprosy patients with subclinical neuropathy will be randomly divided into an intervention group and a placebo group. Newly registered MB and PB patients without clinical NFI but having subclinical sensory and/or motor neuropathy at diagnosis, or developing this within their first three months of MDT treatment, will be eligible for inclusion in the trial. Patients from the Subclinical trial developing NFI in the first three months of the trial can enter the Clinical trial, but only data of patients that were allocated to the placebo group of the Subclinical trial will be analyzed.

#### Inclusion and exclusion criteria

Patients included in the trials should be between 15 and 60 years of age, must give informed consent and should be free from conditions that may affect the peripheral nervous system, such as diabetes mellitus, and other active underlying diseases for example hypertension, osteoporosis and tuberculosis. Included patients receive deworming treatment with Mebendazol before steroid treatment starts. In both studies patients will be followed-up for 18 months. Excluded will be women pregnant at diagnosis, patients who need steroids for reasons other than recent NFI and patients with a single skin lesion on the trunk as the only sign of leprosy.

#### Randomization and blinding

In both trials patients will be randomly allocated to one of the two study arms. Randomization tables are provided by the statistician and drugs are labeled accordingly by the manufacturer. The key is held by the International Coordinator, Study Manager and PI of each centre and will be broken after the data analysis is completed or earlier for patients with adverse events, and new or worsening NFI.

#### Sample size calculation

For the Clinical trial a recovery of 60% is presumed for the standard regimen in the control group [17]. To detect a recovery of 70% in the treatment group, compensating for 20% of loss to follow-up, a total of 720 subjects need to be enrolled.

The INFIR study shows that of the leprosy patients having subclinical neuropathy 16% will eventually develop clinical NFI [1]. For the Subclinical trial we assume a reduction in patients developing clinical NFI by half in the treated group (8%). Anticipating a loss to follow-up of 20% at 18 months, a sample size of 275 subjects per trial arm is required. For both sample size calculations a one-tailed alternative hypothesis, 80% power and 5% significance is used.

#### Follow-up

Patients will be followed-up for 18 months. Nerve function is monitored by monthly VMT and MFT in both trials and assessments with NCS and WDT takes place at 20 weeks, 12 and 18 months from intake in the Subclinical trial. Participants can be withdrawn from the trials because of medical or clinical reasons or pregnancy. Subjects missing their appointment will be visited at home by research staff within two weeks when contact by cell phone is not possible or did not result in late attendance at the clinic. In cases when only one appointment is missed and medication is not taken for a maximum of one month, treatment will continue from the first package missed. The TENLEP trials started in April 2012, with a duration of intake of 1.5 years.

### Medication

In both trials the medication provided is prednisolone. Prednisolone will be allocated based on two bodyweight classes. The low weight class (patients below 50 kg), will receive prednisolone treatment based on a body weight of 45 kg. The high weight class (patients 50 kg and more) receives treatment based on a body weight of 60 kg.

#### Treatment arms

**Figure 1:**
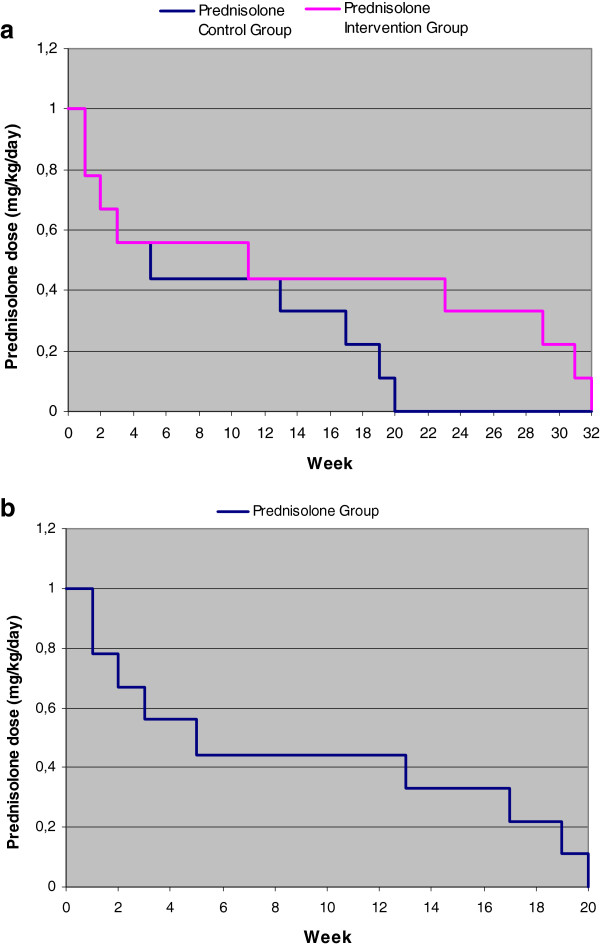
**a. Timeline prednisolone dose for patients under 50 kg in the Clinical trial . b**. Timeline prednisolone dose for patients under 50 kg in the Subclinical trial.

In the Clinical trial patients in the treatment arm receive prednisolone for 32 weeks, in tablets of 5 mg. The control arm will follow a regimen of 20 weeks and receive placebo tablets to keep the number of tablets equal to the treatment group for effective treatment time and duration. The dose in both intervention and control arm starts at 1 mg/kg/day (either 45 or 60 mg/day depending on weight class) and will be tapered down over 32 and 20 weeks, respectively. Figure 1a shows the dose over time of both arms in the Clinical trial. In contrast to previous trials, the middle range of the prednisolone dose will be maintained at a high level (0.44 mg/kg/day) for a longer period (12 weeks). In the Subclinical trial patients receive either prednisolone or placebo for 20 weeks in tablets of 5 mg. The prednisolone dose starts at 1 mg/kg/day (either 45 or 60 mg/day depending on weight class) and will be tapered down over 20 weeks. Figure 1b shows the timeline and dosage. The total dosage of prednisolone over 20 weeks will be 2.8 grams for patients under 50 kg body weight, and 3.7 grams for patients over 50 kg body weight.

#### Adverse events

Prednisolone has known adverse events, ranging from mild adverse events, such as moon face, fungal infections and acne, to serious adverse events such as peptic ulcer, osteoporosis glaucoma, cataract, psychosis, diabetes and hypertension [18,19]. Serious adverse events have not been encountered frequently in previous trials studying prednisolone for treating NFI in leprosy patients [20], however strict monitoring of adverse events will take place in the TENLEP trials. In case of serious adverse effects the PI will break the key and initiate an individualized treatment scheme. In the event of minor side effects additional medication will be prescribed according to normal protocol in the clinic, but the key will not be broken.

### Data collection

#### General assessments

Before intake, the general health of all possible eligible patients will be checked and history will be taken according to the protocol. Additionally, patients will be tested for specific medical conditions related to neuropathy and prednisolone intake such as diabetes mellitus (urine test) and osteoporosis (FRAX). Leprosy status will be categorized using both WHO classification (PB/MB), and Ridley-Jopling classification [21]. The latter will be done on clinical grounds, but a skin biopsy can be taken on voluntary basis for confirmation of the classification by a trained pathologist. For this procedure an additional consent procedure is in place. Of each patient a slit skin smear will be taken for determining the Bacteriological Index (BI).

#### Clinical nerve function tests

After intake, eligible subjects will be assessed for clinical sensory and motor nerve impairment. The clinical sensory function is tested with monofilaments using a standard set of 5 Semmes-Weinstein monofilaments ranging from blue (200 mg) to pink (300 g) [22]. This test of touch sensibility is based on indenting the skin surface with a series of standard nylon filaments. For each thickness it is recorded whether or not the patient feels the touch, starting with the thinnest filament. For hands the normal threshold is the blue filament and for feet the purple (2 mg) filament; when these filaments are felt by the patient it results in a score of 0. The score increases with 1 for each thicker filament not felt, with a maximum score of 5 in hands and 4 in feet (filament of 300 g not felt) for every site. Each nerve will be tested on 3 sites. The trigeminal nerve is tested by assessing the blink regularity. A patient is diagnosed as having sensory impairment if the monofilament threshold is increased by three or more monofilament levels in one single nerve (over 3 sites).

Motor nerve impairment will be assessed with Voluntary Muscle Testing (VMT) using the 0–5 MRC scale [23,24]. The test is performed by checking the ability of the patient to move a body part to a given position and to hold that position against resistance applied by the tester. A nerve scoring lower than 5 is considered impaired. When a single score of 4 is found this score should be independently confirmed by a second assessor.

#### Subclinical nerve function tests

When new patients show no abnormal values by VMT and MFT they will be tested further for possible inclusion in the Subclinical trial. For detecting subclinical neuropathy sensory and motor Nerve Conduction Studies (NCS), carried out with the Neurocare 2000® EMG machine (BioTech Ltd, Mumbai), and Warm Detection Threshold (WDT) test, using the TSA II (MEDOC, Israel), will be performed. NCS and WDT will take place in an air-conditioned room at approximately 20-25°C.

All assessments will be carried out at both sides of the body. Table 1 shows which nerves will be tested and methods used. MFT and VMT will be assessed at start of the trial and subsequently every month in both studies and follow-up periods on all subjects. WDT, Sensory Nerve Conduction (SNC) and Motor Nerve Conduction (MNC) will be carried out at baseline, end of treatment period (20 weeks) and at 12 and 18 months. For the Clinical trial additional information will be obtained using the Reaction Severity Scale (RSS) [25], the Screening of Activity Limitation and Safety Awareness (SALSA) scale [26] and the Participation Scale [27]. All scales will be filled in at baseline, end of treatment period (32 weeks), 12 and 18 months.

**Table 1:** Nerves assessed by the different methods

**Nerve**	**VMT**	**MFT**	**MNC**	**SNC**	**WDT**
Trigeminal		blink			
Facial	x				
Ulnar	x	x	x	x	x
Median	x	x	x	x	x
Radial	x	x		x	x
Common peroneal	x		x		
Post. Tibial	x	x	x		x
Sural		x		x	x

#### Reliability and normative studies

Prior to intake inter-tester reliability studies have taken place for all assessments (MFT, VMT, WDT, NCS) to test and improve comparability of the results and ensure high measurement quality. After reliability studies for NCS and WDT, normative studies were carried out to establish the local normal values for each separate collaborative centre. For each nerve a minimum of 150 normal subjects from surrounding areas were tested, equally spread over both sexes and 3 age categories (15–30, 31–45 and 46–60 years of age). Normal subjects will be screened to ascertain they do not have diabetes or nerve function impairment.

#### Standard operating procedures and training

For all assessments standard operating procedures (SOP) have been developed. To achieve consistency of the assessments between all research centers, the PI and two main assessors of each centre received training in research protocol procedures and handling equipment. In addition, an online Good Clinical Practice (GCP) course was completed by all PI’s.

### Outcome measures

#### The Clinical trial

**Figure 2:**
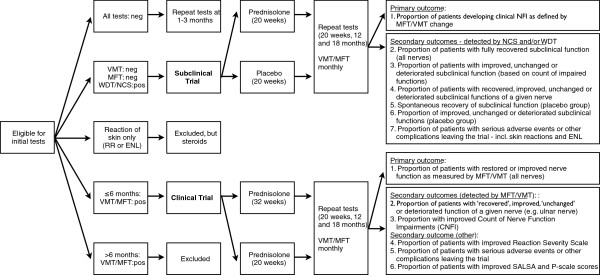
Overview of intake, assessments and outcomes in the Clinical and Subclinical trial.

The primary study outcome is the proportion of patients with restored and improved nerve function (of all nerves) measured by VMT/MFT at 18 months. Secondary outcomes are based on results of the Reaction Severity Scale, SALSA and Participation Scale (Figure 2). Furthermore, a Count of Nerve Function Impairment (CNFI) will be computed to measure the results of treatment on nerve function, with special interest for most commonly affected nerves. The CNFI will consist of the count of nerve function impairments detected by monofilament and VMT testing and will be validated in this trial.

#### The Subclinical trial

The proportion of patients developing clinical NFI as measured by MFT and VMT is the primary outcome indicator (Figure 2). Secondary outcome measures focus on results of the specific assessments for detection of subclinical neuropathy (WDT, SNC and MNC).

### Data analysis

After the data is entered for each centre, data will be checked and cleaned. Regular weekly back-ups will be made for local off-site storage in addition to a monthly upload of data via a web-based repository to the project statistician.

#### Reliability studies

Inter-rater reliability for categorical outcomes of VMT and MFT are assessed using weighted Kappa statistics. Comparing differences and averages of paired assessments for WDT, SNC and MNC tests are based on Bland-Altman plots. In addition, a mixed model analysis of variance will be used to compute the intraclass correlation coefficient.

#### Normative studies

To determine normative values for WDT and NCS for each centre, outliers are excluded before limits of normal function are calculated using an approach based on regression analysis taking into account age-related trends. The 97.5^th^ percentile is used to define abnormal function.

#### Primary outcomes

Analysis of primary outcomes will be done at 20 (Subclinical trial) or 32 weeks (Clinical trial), 12 and 18 months. Possible effects of potential covariates (gender, age, leprosy classification) will be assessed with ANCOVA. All analyses will be carried out using Stata statistical software.

#### Secondary outcomes

Continuous outcome measures will be analyzed with Analysis of (co)variance, assessing one or more covariates. Categorical outcomes may be analyzed using chi-squared test or log linear models. For Survival outcomes the log rank test will be used.

### Ethics

All national Research Ethics Committees have approved the study protocol, which are for India: Indian Council of Medical Research; Nepal: Nepal Health Research Council (NHRC); Indonesia: Komite Etik Penelitian Kesehatan RSUD Dr. Soetomo Surabaya; Bangladesh: Bangladesh Medical Research Council- National Research Ethics Committee. In addition all local Research Ethics Committees have given their approval as well. Written consent will be obtained from individual subjects before inclusion and for minors additional consent from their guardians will be sought.

## Discussion

This paper describes the protocols of two randomized controlled clinical trials within the TENLEP study. In the Clinical trial the effect of long term prednisolone treatment on the restoration of clinical NFI will be investigated. In the Subclinical trial the efficacy of prednisolone in preventing the development of clinical NFI, in patients with subclinical neuropathy, will be examined.

The optimal dose and duration of prednisolone for treating clinical NFI in leprosy has not been established yet and there is not enough evidence available from randomized controlled clinical trials on the long term effect of prednisolone treatment [3]. The TENLEP study addresses this knowledge gap and will extend the current understanding of prednisolone regimens for the treatment of clinical NFI by comparing a prednisolone treatment of 32 weeks and 20 weeks under controlled circumstances. If a 32 weeks treatment proves to be effective in restoring or improving clinical NFI in leprosy patients, new guidelines can be developed which can significantly improve patient management in leprosy care and especially a positive effect on the prevention of disabilities (POD) can be expected.

This study will be the first to evaluate prednisolone treatment for the prevention of clinical NFI in people affected by leprosy diagnosed with subclinical neuropathy. The results of the preceding TRIPOD trial provide some insight in possible effects of prednisolone in preventing new NFI [28]. To prevent new NFI and reactions, leprosy patients with and without pre-existing NFI at diagnosis (as determined with VMT and MFT) received a prophylactic low dose of prednisolone (20 mg/day) for four months, tapered down in the last month. Although a reduced incidence of new NFI and reactions was observed at the end of treatment at four months, this was not sustained at one year. More important however, the preventive effect of prednisolone at four months was more than three times higher in patients with no pre-existing NFI [28]. A second study that has some similarities with our Subclinical trial is of that of Capadia et al. [29], who studied the effect of prednisolone on neuropathy as assessed with NCS. In their study, neuropathy was divided in mild, moderate and severe groups, as percentages deviating from normative values. Their findings suggest that a 12-week prednisolone course is not effective in preventing or reversing nerve damage. However, they found that mildly affected nerves showed higher improvement rates than moderately and severely affected nerves (53%, 21% and 14% of the nerves improved respectively).

The better outcomes of prednisolone treatment and prophylaxis on non-affected and mildly affected nerves from both studies of TRIPOD and Capadia et al. [29] show the importance of early treatment of neuropathy in leprosy patients. With the Subclinical trial we hope to establish that prednisolone treatment can prevent the development of clinical NFI and in this way can prevent disabilities and deformities in newly diagnosed leprosy patients.

If the prednisolone treatment turns out to be effective in the prevention of clinical NFI, the implementation of treatment for patients with evident subclinical neuropathy in clinical practice will be complicated. The methods to detect subclinical neuropathy used in this study (TSA II and Neurocare 2000) will not be available in the field, since the devices are expensive and conditions under which the assessments have to take place, a steady environmental temperature of 20–25°C, are difficult to realize in tropical climates. Therefore it is important to search for a cheaper, portable method to detect subclinical neuropathy that can be easily used in field clinics.

However, also without a test to determine subclinical neuropathy the results of this study can be useful to improve treatment guidelines. The current prediction rule allows to distinguish leprosy patients with a high risk for developing NFI [30]. However, at the moment, providing prophylactic prednisolone treatment to this group of patients is considered unethical, as a significant number of patients will take prednisolone unnecessarily. With the information of the TENLEP trials we hope the prediction rule can be refined, so administering prophylactic prednisolone is acceptable in certain, well defined groups.

In conclusion, the TENLEP study will add to the current understanding of neuropathy due to leprosy and will provide better insight into the effectiveness of prednisolone treatment in the prevention and recovery of nerve function loss in leprosy patients. If this study shows the effectiveness of prednisolone in the prevention and recovery of NFI it will improve treatment options and contribute therefore to the prevention of permanent sensory and/or motor nerve function loss in people affected by leprosy and hence prevent disabilities and deformities which will improve the lives of many of these patients.

## Abbreviations

BI: Bacteriological Index; CNFI: Count of nerve function impairment; GCP: Good clinical practice; ISC: International steering committee; MB: Multi bacillary; MDT: Multi drug treatment; MFT: Monofilament test; MI: Motor impairment; MNC: Motor nerve conduction; MRC: Medical research council; NCS: Nerve conduction study; NFI: Nerve function impairment; PB: Paucibacillary; PI: Principal investigator; POD: Prevention of disability; RCT: Randomized controlled trial; RSS: Reaction severity scale; SALSA: Screening of activity limitation and safety Awareness; SI: Sensory impairment; SNC: Sensory nerve conduction; SNFS: Subclinical nerve function Score; TENLEP: Treatment of early neuropathy in LEProsy; VMT: Voluntary muscle test; WDT: Warm detection threshold.

## Competing interests

The authors declare that they have no competing interests.

## Authors’ contributions

EP, WvB, WB, IW and PN designed the study and drafted the protocols. IW wrote the manuscript. DL, PS, CS, EWS, and JHR participated in the design of the study and all authors revised the manuscripts. All authors have read and approved the final manuscripts.

## Funding

This research is funded by the American Leprosy Mission, German Leprosy and TB Relief Association, Netherlands Leprosy Relief, Ordre de Malte, and the Turing Foundation.

## Pre-publication history

The pre-publication history for this paper can be accessed here:

http://www.biomedcentral.com/1471-2377/12/159/prepub
